# Human hair genealogies and stem cell latency

**DOI:** 10.1186/1741-7007-4-2

**Published:** 2006-02-03

**Authors:** Jung Yeon Kim, Simon Tavaré, Darryl Shibata

**Affiliations:** 1 Department of Pathology, University of Southern California Keck School of Medicine, Los Angeles, CA 90033, USA; 2 Department of Biological Sciences, University of Southern California, Los Angeles, CA 90089, USA; 3 Department of Oncology, University of Cambridge, Cambridge, UK; 4 Department of Pathology, Inje University Sanggye-Paik Hospital, Sanggye 7 dong 761-7, Nowon-gu, Seoul, Korea

## Abstract

**Background:**

Stem cells divide to reproduce themselves and produce differentiated progeny. A fundamental problem in human biology has been the inability to measure how often stem cells divide. Although it is impossible to observe every division directly, one method for counting divisions is to count replication errors; the greater the number of divisions, the greater the numbers of errors. Stem cells with more divisions should produce progeny with more replication errors.

**Methods:**

To test this approach, epigenetic errors (methylation) in CpG-rich molecular clocks were measured from human hairs. Hairs exhibit growth and replacement cycles and "new" hairs physically reappear even on "old" heads. Errors may accumulate in long-lived stem cells, or in their differentiated progeny that are eventually shed.

**Results:**

Average hair errors increased until two years of age, and then were constant despite decades of replacement, consistent with new hairs arising from infrequently dividing bulge stem cells. Errors were significantly more frequent in longer hairs, consistent with long-lived but eventually shed mitotic follicle cells.

**Conclusion:**

Constant average hair methylation regardless of age contrasts with the age-related methylation observed in human intestine, suggesting that error accumulation and therefore stem cell latency differs among tissues. Epigenetic molecular clocks imply similar mitotic ages for hairs on young and old human heads, consistent with a restart with each new hair, and with genealogies surreptitiously written within somatic cell genomes.

## Background

**Figure 1:**
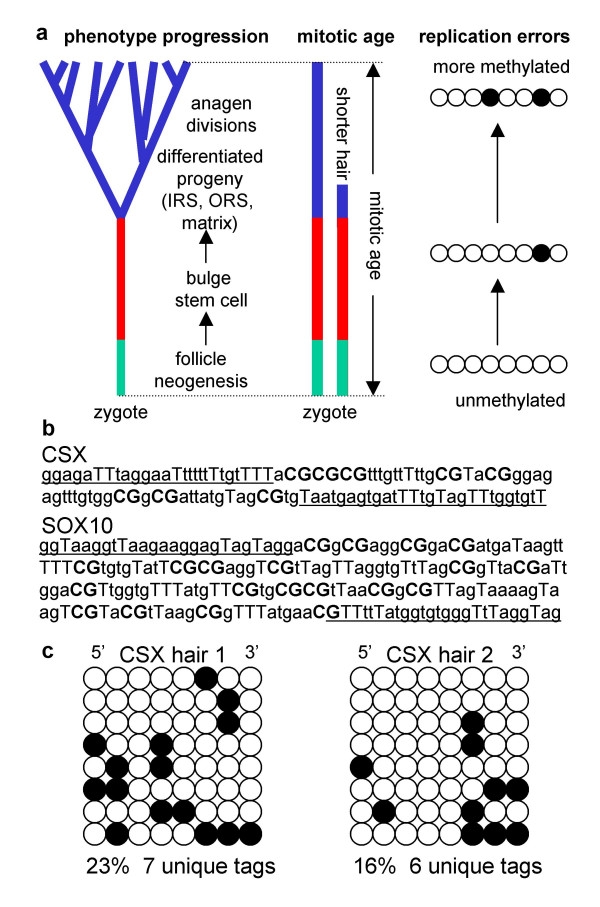
**Human hair genealogy**. **a**. Hair follicle genealogy can be divided into three phenotypic phases: neogenesis, bulge stem cell latency, and anagen differentiation and division. Mitotic age includes divisions during all three phases and may be inferred from numbers of replication errors (methylation) in sampled follicle cells. Short hairs should have fewer anagen divisions relative to longer hairs and therefore fewer errors (less methylation). Hairs from individuals of different ages have similar neogenesis and anagen intervals, and therefore any age-related increase in average methylation reflects lifelong stem cell mitotic activity. Average mitotic ages would be similar for hairs plucked from different aged individuals because of stem cell bulge latency. **b**. Sequences (after bisulfite treatment) of the CSX and SOX10 methylation tags. CpG sites that may be methylated are in bold, and converted Cs in non-CpG sites are capital T's. PCR primers are underlined. **c**. Examples of CSX tags sampled from two follicles (eight tags per hair) from a 37 year old. Circles represent the 5' to 3' order of adjacent CpG sites and filled circles are methylated.

One way to organize the billions of cells within an individual is through genealogy, because all cells are related. Each cell has its own genealogy, which starts from the zygote and ends with the current phenotype. Conceptually, the genealogy of a differentiated epithelial cell can be divided into three distinct phases: neogenesis or development between the zygote and tissue formation, stem cell latency, and differentiation (Figure 1). Stem cells are the progenitors or common ancestors of the much larger numbers of differentiated cells that compose our bodies. The stem cell phase is chronologically the longest because developing and differentiated cells survive for relatively short periods. However, it is uncertain how often stem cells divide because they are rare, difficult to culture, and lack unique identifying markers.

Stem cells are few in number, but it is possible to infer how often they divide by measuring the genomes of their more abundant differentiated descendants. The mitotic age of a differentiated cell can be defined as the total numbers of divisions since conception, which includes the three genealogical phases. Development and differentiation are highly regulated, and numbers of divisions during these phases are similar regardless of age. In contrast, stem cells may divide throughout life. Therefore, any increase in average mitotic age primarily depends on stem cell division.

To apply this logic, we infer mitotic age by measuring the errors that occur during genome replication. The idea is that replication errors surreptitiously record cell divisions, the number of errors being proportional to the number of divisions. Such molecular clock approaches are commonly employed to trace species ancestry [1], but conceivably somatic cell genealogies are also recorded within genomes. Sequences are initially similar in all cells but will become increasingly polymorphic owing to rare and random replication errors. In theory, ancestry may be inferred from polymorphisms sampled from any population, including somatic cells. Sequences rarely mutate, but epigenetic errors also exhibit somatic inheritance and methylation measurably accumulates at certain CpG-rich regions during normal human aging [2,3]. The 5' to 3' order of CpG methylation provides binary information strings (i.e. "0" for unmethylated and "1" for methylated) or tags analogous to sequences, which appear to function as somatic molecular clocks [3]. In general, percent methylation is equivalent to mitotic age because our tags, like most CpG islands [4], are initially unmethylated.

Human intestinal studies revealed an age-related increase in tag methylation, suggesting that crypt stem cells divide throughout life [3,5]. Although cell division appears to be required for de novo in vitro CpG island methylation [6], some in vivo methylation could also occur in non-dividing cells. Although it is difficult to test directly whether tag methylation primarily represents replication errors, such errors probably occur in all cells and should record genealogies in many tissues. Human hair can provide a further test of such an epigenetic molecular clock because its biology suggests much greater stem cell latency.

Hair is periodically shed because follicles exhibit cycles of growth (anagen), degeneration (catagen) and rest (telogen). Differentiated cells including the matrix and inner root (IRS) and outer root sheath (ORS) keratinocytes are shed when the lower follicle physically disappears during catagen [7,8]. A new hair cycle is thought to be initiated after telogen from stem cells located in the bulge. According to a bulge activation hypothesis [9], stem cells briefly divide at the start of anagen to produce differentiated progeny that reconstruct and maintain a new lower follicle. Most divisions occur in differentiated cells that die at the end of anagen whereas stem cells seldom divide and survive each cycle.

This biology suggests hair genealogies with relatively few stem cell divisions, since most divisions occur in differentiated cells during anagen, and greater mitotic ages in longer hairs. Variations among hairs on a single individual will primarily reflect differences in anagen divisions because lower follicle mitotic activity is high, cycles are asynchronous, and anagen lasts a variable number of years [8,10]. Age-related methylation should be minimal to undetectable because of relative bulge stem cell quiescence. However, hair biology has mainly been studied in mice, and human anagen cycles are longer (years versus weeks) and usually recur for decades. Relatively little is known about human hair stem cell dynamics because experimental manipulations are impractical.

## Results

### Average hair methylation does not increase beyond infancy

Seven scalp hairs were randomly plucked from the vertex of 28 individuals of different ages. The plucked hairs were primarily in anagen and contained differentiated IRS and ORS cells, though some matrix cells may be present [10,12]. Methylation patterns were sampled from each hair using bisulfite sequencing of two CpG rich regions or tags (Figure 1). One tag called CSX contains 8 CpG sites in the 3' untranslated region of the CSX gene and has been used to study the intestines [3]. The other tag is in the promoter region of the SOX10 gene and contains 20 CpG sites.

**Figure 2:**
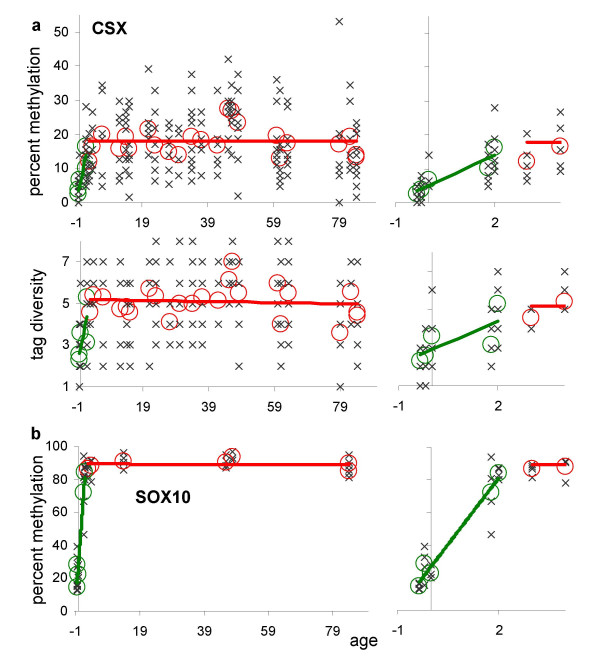
**Methylation with age**. **a**. CSX tags sampled from anagen follicles. Individual hair values ("X") were scattered but averages ("O") from individuals exhibited no increase after two years of age. Tag diversity (number of unique tags found in the 8 sampled follicle tags) also exhibited an increase before two years of age. Graphs on the right give an expanded view of values early in life. The earliest hairs are from fetuses of 22 and 32 weeks estimated gestational ages, and a term stillborn. Symbols and trend lines are green up to two years of age, and red after infancy. **b**. Average SOX10 methylation also exhibited relatively constant values after two years of age.

Methylation patterns were polymorphic between and within hairs (Figure 1). Values for individual hairs were scattered, but average methylation was low before birth, progressively increased until two years of age, and then was constant (Figure 2). The lack of age-related increase in hair methylation is consistent with infrequently-dividing stem cells because the average numbers of divisions during neogenesis and anagen are likely to be similar for all hairs. Follicle populations were polymorphic with CSX tag diversity (number of unique patterns per eight sampled tags) rising to an average of about 5.5 unique tags in adulthood. SOX10 is a "faster" molecular clock than CSX because it becomes nearly fully methylated by about two years of age (Figure 2). Hair after two years of age had average SOX10 methylation above 80%, and only a single tag (1 of 234 (0.4%)) was unmethylated.

### Average CSX hair methylation varies during the hair cycle

**Figure 3:**
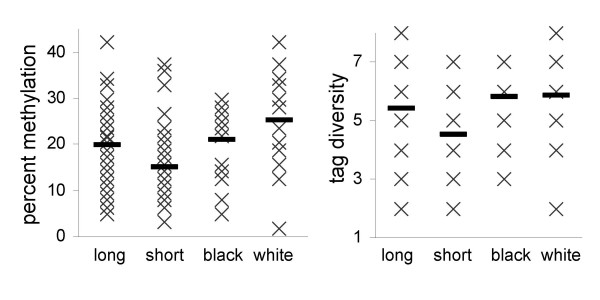
**Summary of CSX tag methylation and diversity between different hair phenotypes**. Average hair methylation increased from short to long hairs, and there were no significant differences between pigmented and white hairs. Tag diversity was significantly lower for short hairs. (Bar is overall average and "X" indicates individual hairs.)

Hair length was employed as a surrogate for anagen cycle length. CSX methylation patterns were compared between long (>40 cm) and short (<4 cm) hairs (Figure 3). There was no direct relationship between hair length and methylation, some long hairs having very low methylation levels and some short hairs having very high ones. However, average methylation was significantly higher for long (19.8%) versus short (14.9%) hairs (Figure 3 and Table 1). Tag diversity values were also scattered, but average diversity was also significantly greater in long hairs. SOX10 percent methylation was also greater in random compared to short hairs (Table 1).

### Average methylation is not significantly different between pigmented and white hairs

Methylation was compared between white and pigmented hairs from three individuals (Figure 3). There were no significant differences, suggesting similar cell kinetics (Table 1). SOX10 is important for melanin production [13] and the extensive SOX10 promoter methylation (>80%) in pigmented and white hair implies lack of expression. To test whether SOX10 is methylated in melanin producing cells, we examined three cell lines. SOX10 was unmethylated (<2%) in two pigmented melanoma cell lines (M624 and M888) and methylated (95%) in a breast cancer cell line (MCF7). CSX was extensively methylated (>90%) in all three tumor cell lines, consistent with multiple divisions before and after transformation.

### Validation and reproducibility

**Figure 4:**
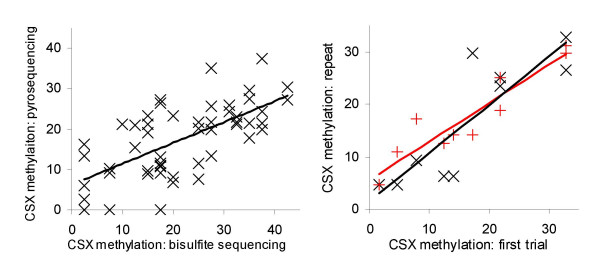
**Validation and reproducibility**. **a**. Comparison between bisulfite sequencing and pyrosequencing for the first five CSX CpG sites with different hairs. Correlation coefficient is 0.65. **b**. Comparisons of bisulfite sequencing of eight more bacterial clones from the same PCR (black "X's" and trend line) or from repeat PCR of the same hair (red crosses and trend line). Identical methylation patterns were not obtained with resampling, probably because hair follicles contain complex mixtures of alleles, but correlation coefficients are respectively 0.86 and 0.93.

The scatter of the methylation data probably reflects the stochastic nature of replication errors, cell division, survival and migration. However, it is uncertain if bisulfite sequencing of eight alleles adequately samples follicle methylation. To help validate our methodology, methylation of the first five CSX CpG sites was also measured by pyrosequencing [11], which should average methylation from greater numbers of PCR products. Measurements were similar with pyrosequencing and bisulfite sequencing (correlation coefficient of 0.65), although percent methylation was generally greater with bisulfite sequencing (Figure 4A).

Reproducibility was tested by resampling eight more bacterial clones from the same PCR/cloning experiment, or by repeating the PCR on the same hair and sampling eight more clones (Figure 4B). Although methylation patterns were not identical after resampling, average hair percent methylation values were similar (correlation coefficients of 0.86 for resampling the same PCR/cloning and 0.93 for repeat PCR of the same hair). Therefore, a sample of eight alleles appears to approximate the complex methylation patterns likely to be present in a human hair follicle.

### Discussion

Human cell fate studies are hampered by the inability to mark and follow individual cells experimentally. Here we employ a molecular clock strategy to reconstruct human hair genealogies from random epigenetic replication errors. Contemporary sequences are routinely used to reconstruct species ancestry, with times since a common ancestor proportional to numbers of sequence differences [1]. By analogy, all cells are related through a single, continuously expanding somatic cell tree, and stem cells are common ancestors (Figure 1). For example, the zygote is the stem cell or common ancestor for all somatic cells, and a hair stem cell would produce differentiated follicle cells. Depending on genealogy, there may be multiple common ancestors or stem cells per follicle. Hair provides a well-defined empirical test of an epigenetic molecular clock because its genealogy would be consistent with only a limited number of potential methylation patterns.

The seemingly "random" methylation tag patterns observed in hair (Figure 1) are consistent with stochastic replication errors. Some of the scatter of average hair values is probably secondary to the small number of alleles sampled per hair, but control experiments illustrate reproducibility (Figure 4B). The ability to sample reproducibly a human hair follicle containing thousands of cells with just eight tags may reflect that follicle cells are closely related to each other. The lack of an age-related increase in average hair methylation is consistent with the bulge activation hypothesis [9], in which stem cells primarily divide at the start of anagen to produce progeny that reconstruct and maintain a new lower follicle. Replication errors in differentiated cells cannot accumulate because they are discarded when the lower follicle degenerates after several years. A new cycle begins after several weeks, and new hairs in young or old individuals would have similar mitotic ages if bulge stem cells are normally latent and only divide a few times every cycle.

Hair neogenesis or development precedes the stem cell phase. Human follicle neogenesis starts late in the first trimester [14,15]. Stem cells appear to originate from rapidly dividing hair peg germ cells that grow fine lanugo hairs, which fall out around birth to be replaced by adult-type terminal scalp hairs. Consistent with the idea that bulge stem cells are descendents of mitotic germ cells, methylation rapidly increased from low levels before birth. Average tag methylation attained maximum or adult average values about two years after birth (Figure 2), suggesting bulge stem cell latency is not fully established until after infancy. SOX10 is a faster clock than CSX, and the lack of unmethylated SOX10 tags in older individuals suggests that most bulge stem cells had previously divided before becoming latent after infancy. High mitotic activity followed by latency would allow for the label-retaining hallmark of bulge stem cells.

To characterize the differentiated phase, hair length was employed as a surrogate for anagen cycle length. Consistent with new hairs emerging from descendants of infrequently dividing bulge stem cells, average CSX methylation was significantly lower for short versus long hairs and average diversity was also significantly lower in short hairs, suggesting a relative population bottleneck. However, there was no consistent relationship because some long hairs had very low methylation levels and some short hairs had very high ones. This variation may reflect the inability definitively to select hairs of different lengths, which can be confounded by hair breakage (short hairs may be broken long hairs), cycle length heterogeneity between hairs, and the variable cellular compositions of plucked hairs [10]. Variation in methylation with respect to hair length may also reflect anagen bulge cell migration. Transplanted bulge progeny in whiskers migrate down the ORS throughout anagen [16], which could counteract a steady increase in average anagen hair mitotic age if these immigrants have lower mitotic ages. Anagen bulge migration may not necessarily require new stem cell divisions because these cells may originate from the suprabasal bulge, a region with the early progeny of basal bulge stem cells [17]. In addition, cells from the previous anagen cycle (secondary hair germ) may survive through telogen and help reconstitute the new follicle [18].

White hairs were slightly more methylated than pigmented hairs from the same heads, possibly suggesting more lifetime divisions in white hairs. However, this difference was not statistically significant and further studies are necessary to determine whether mitotic ages differ between pigmented and white hairs. SOX10 is important for melanin production [13] and the SOX10 promoter methylation (>80%) in both pigmented and white hair, but not in melanoma cell lines, implies lack of expression [19]. The melanocyte lineage appears to be infrequent in plucked hairs because only one of 234 SOX10 tags sampled after two years of age was unmethylated like the melanoma cell lines. Therefore, consistent with separate stem cell origins for melanocytes and other follicle cells [20], epithelial lineages appear distinct from the melanocyte lineage.

**Figure 5:**
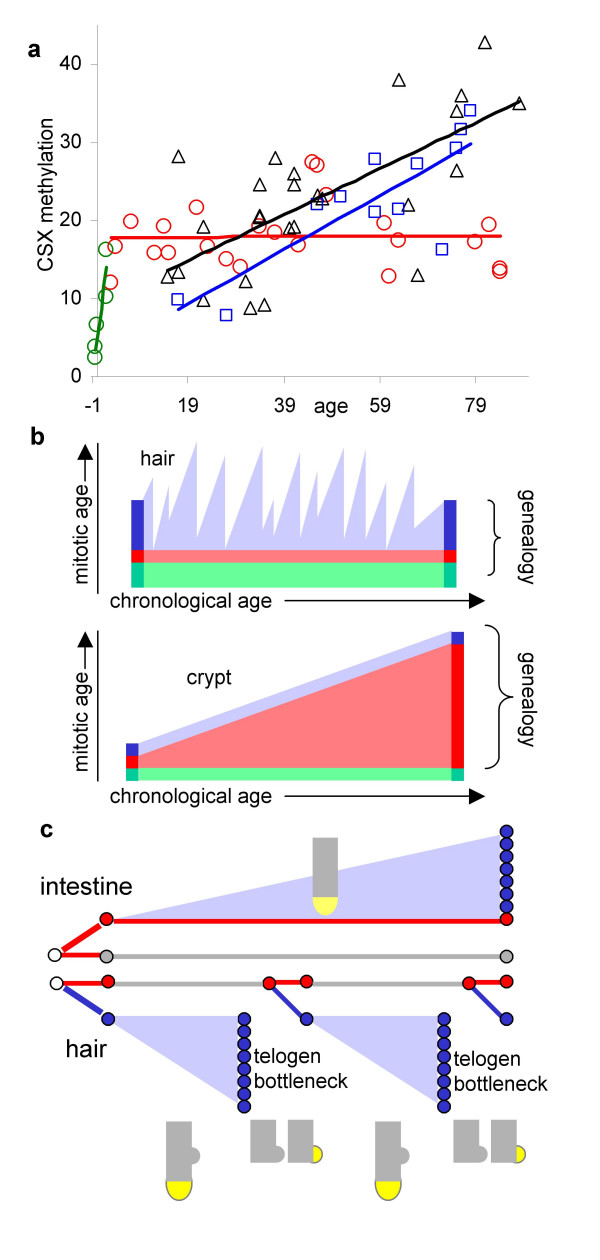
**The CSX molecular clock across different human tissues**. **a**. Comparisons of CSX tag methylation between human hair (green or red), small intestines (blue) and colon (black). Intestinal data [3, 5, 21] include additional unpublished individuals. Plotted are average values from each individual. In contrast to hair, the intestines show an age-related increase in methylation. Regression analysis of intestinal data were consistent with positive slopes (F_1,25 _= 21.58, P < 0.0001 for the colon, and F_1,10 _= 21.03, P = 0.001 for the small intestines), but not with hair data after two years of age (F_1,21 _= 0.0009, P = 0.98). **b**. Hair and intestinal genealogies can be divided into three phases (neogenesis (green), stem cell (red) and differentiation (blue)). Anagen cycles allow for episodic changes in hair mitotic ages due to lower follicle divisions, but average mitotic age remains constant with chronological age because bulge stem cell divisions are infrequent. Differentiated intestinal cells divide only a few times and survive about a week, and therefore the age-related increase in mitotic age represents crypt stem cell divisions. Crypt stem cells appear to divide more often than bulge stem cells. **c**. Hair has two distinct mitotic compartments (lower follicle and bulge, indicated in yellow) whereas the intestines have a single mitotic compartment at the crypt base. The lower follicle physically disappears at telogen and previously mitotic lineages become extinct. This telogen bottleneck allows infrequently dividing bulge stem cells to become common ancestors because their progeny repopulate the new lower follicle. The telogen bottleneck effectively resets the mitotic age of the follicle. A similar bottleneck does not occur in intestinal crypts and therefore a cell must divide frequently to become a common ancestor. Without a bottleneck, crypt mitotic age increases with age. A quiescent crypt cell may have stem cell potential, but is not the common ancestor for present day differentiated cells.

Cell divisions and therefore replication errors occur in many tissues, and by analogy to species molecular clocks, a reliable somatic cell molecular clock should allow comparisons among tissues. CSX tags have been previously sampled from human large and small intestinal crypts [3,5,21], which are also clonal structures [22]. In contrast to hair, intestinal methylation increased throughout life, with average levels exceeding hair later in life (Figure 5). Intestinal methylation is also primarily sampled from differentiated cells and therefore its genealogy can be divided into three phases (neogenesis, stem cell and differentiation). In contrast to years of anagen, differentiated crypt cells only divide a few times and survive about a week. As in hair, average neogenesis and differentiation intervals are similar regardless of age, and therefore the age-related increase in intestinal methylation suggests crypt stem cells divide more often than bulge stem cells. Alternatively, other factors such as differences in environment or subtle differences in low-level gene expression (CSX is normally expressed only in the heart [23]) may influence how methylation changes with age. However, a recent study of human endometrium was also consistent with replication errors as a source for age-related CSX methylation because percent methylation correlated with likely numbers of menstrual cycles [24].

Niche structure may help explain different mitotic ages (Figure 5). Intestinal mitotic compartments, located at crypt bases, are preserved throughout life. Although most mitotic intestinal cells are considered to be transit amplifying rather than stem cells [22], dividing cells may persist as long as their mitotic compartment or niche persists. Without a bottleneck mechanism to eliminate mitotic cells, they may accumulate errors throughout life and effectively function as stem cells or common ancestors because they produce the majority of cells within a crypt. By contrast, the primary hair mitotic compartment (lower follicle) is physically destroyed every few years, which eliminates most dividing cells and their accumulated errors. Quiescent bulge stem cells can function as common ancestors because they briefly divide and replenish the lower follicle at critical periods after such bottlenecks. Configurations physically differ between stem cell niches [7], which could influence the genealogies of their tissues.

## Conclusion

Age-related changes in methylation or mitotic ages appear to depend on how often stem cells divide. Although the stochastic nature of replication errors may hinder the interpretation of molecular clocks, and many questions remain [25], such approaches provide information that is difficult to obtain by other more precise methods. Further studies are needed to define human somatic molecular clocks better, and more information can be extracted with more data and formal quantitative modeling. Here we infer human stem cell genealogies from present day methylation patterns consistent with biology reconstructed by more direct methods, primarily in mice. Epigenetic molecular clocks are experimentally much more efficient than sequence somatic clocks because mutation frequencies in tumor cell lines are about one per million bases [26] whereas multiple epigenetic errors are readily found after sequencing only a few hundred bases (Figure 1). Replication errors are synonymous with division, and somatic molecular clocks that count these errors potentially facilitate the systematic reconstruction of many human tissue genealogies, allowing a fundamental distinction between old and new cells within the same individual.

## Methods

Randomly chosen vertex scalp hair follicles were obtained by plucking from 28 individuals of different ages. This research was approved by the Health Sciences Institutional Review Board at the University of Southern California. Most of these hairs were in anagen because telogen hairs (obtained by pulling rather than plucking [10]) generally did not contain sufficient DNA for amplification (data not shown). For CSX, seven hairs each with eight tags per hair were sampled from each head. For SOX10, four to five hairs, each with six tags per hair, were sampled. Long hairs were greater than 40 cm and short hairs were shorter than 4 cm on the same head. DNA was extracted by placing the follicle in 10 μl of 10 mM Tris-HCl, 2 mM EDTA (pH 8.0) with 20 mg/ml Proteinase K for two hours at 56°C. The solution was boiled for 5 min and DNA was bisulfite converted using an agarose bead method [3]. The final bead volume was about 25 μl. Approximately 10% (2.5 μl) of the bead was amplified with 42 PCR cycles using primers specific for CpG rich regions or tags in the CSX and SOX10 genes (Figure 1). Each follicle was amplified in duplicate and PCR products were combined prior to cloning (TOPO TA Cloning kit, Invitrogen, Carlsbad, CA). Tags with evidence of incomplete bisulfite conversion (Cs at non-CpG sites) were discarded from the analysis. Average hair methylation was defined as the average number of methylated sites among tags from each follicle. Follicle diversity of a hair was defined as the number of unique tags found among the sampled tags.

Methylation of the first five CSX CpG sites was also measured with pyrosequencing [11]. The biotinylated primer was 5' -ACACCAAACTACAAAATCACTCATTA-3' and the pyrosequencing primer was 5' -TAGGAATTTTTTTTGTTTTA-3'. A suitable pyrosequencing primer could not be located for the last 3 CpG sites. The assay was performed on a PSQ HS 96 Pyrosequencing System with conditions recommended by the manufacturer (Pyrosequencing, Inc., Westborough, MA).

## Authors' contributions

JYK performed most of the experiments and helped write the manuscript. ST helped with the statistical and quantitative analysis. DS analysed the data and wrote the manuscript.

## Figures and Tables

**Table 1:** Short, long, pigmented and white hairs

	Long Hair	Short Hair	P*	Pigmented	White	P*
CSX % Methylation	19.8 (56)	14.9 (42)	0.006	21.0 (15)	25.1 (16)	0.23
CSX Diversity	5.4 (56)	4.5 (42)	0.004	5.8 (15)	5.9 (16)	0.88
SOX10 % Methylation	89.8** (20)	84.0 (8)	0.073	89.7 (6)	89.3 (6)	0.87

() numbers of hairs examined

* two sided t-test

**Random hairs examined
